# Epidemiology, prevention, and treatment of new-onset atrial fibrillation in critically ill: a systematic review

**DOI:** 10.1186/s40560-015-0085-4

**Published:** 2015-04-23

**Authors:** Takuo Yoshida, Tomoko Fujii, Shigehiko Uchino, Masanori Takinami

**Affiliations:** Intensive Care Unit, Department of Anesthesiology, The Jikei University School of Medicine, 3-19-18, Nishi-Shinbashi, Minato-ku, Tokyo 105-8471 Japan

**Keywords:** Atrial fibrillation, Critical care, Outcome, Prevention, Treatment, Systematic review

## Abstract

**Background:**

Atrial fibrillation (AF) is a common arrhythmia in the ICU. The aim of this review is to summarize relevant information on new-onset AF in non-cardiac critical illness with respect to epidemiology, prevention, and treatment.

**Methods:**

We conducted a PubMed search in June 2014 and included studies describing the epidemiology, prevention, and treatment of new-onset AF and atrial flutter during ICU stay in non-cardiac adult patients. Selected studies were divided into the three categories according to the extracted information. The methodological quality of selected studies was described according to the Grading of Recommendations Assessment, Development and Evaluation system.

**Results:**

We identified 1,132 citations, and after full-text-level selection, we included 10 studies on etiology/outcome and five studies on treatment. There was no study related to prevention. Overall quality of evidence was mostly low or very low due to their observational study designs, small sample sizes, flawed diagnosis of new-onset AF, and the absence of mortality evaluation. The incidence of new-onset AF varied from 4.5% to 15.0%, excluding exceptional cases (e.g., septic shock). Severity scores of patients with new-onset AF were higher than those without new-onset AF in eight studies, in four of which the difference was statistically significant. Five studies reported risk factors for new-onset AF, all of which used multivariate analyses to extract risk factors. Multiple risk factors are reported, e.g., advanced age, the white race, severity scores, organ failures, and sepsis. Hospital mortality in new-onset AF patients was higher than that of patients without AF in all studies, four of which found statistical significance. Among the five studies on treatment, only one study was randomized controlled, and various interventions were studied.

**Conclusions:**

New-onset AF occurred in 5%–15% of the non-cardiac critically ill patients. Patients with new-onset AF had poor outcomes compared with those without AF. Despite the high incidence of new-onset AF in the general ICU population, currently available information for AF, especially for management (prevention, treatment, and anticoagulation), is quite limited. Further research is needed to improve our understanding of new-onset AF in critically ill patients.

## Background

Atrial fibrillation (AF) is a common arrhythmia in critically ill patients [1-4]. To date, multiple studies on incidence, prophylaxis, and treatment of new-onset AF in specific cohorts have been published, such as cardiac surgery [5-15], non-cardiac surgery [16-23], and medical diseases [24-27]. Especially in cardiac surgery and cardiac diseases, incidence and treatment of AF have been extensively investigated, which have led to publication of many reviews and established recommendations in guidelines [28-35]. Compared with the amount of knowledge for cardiac diseases, however, information on AF occurring in general critically ill populations is limited [36,37].

Another issue related to AF in critically ill patients is their ambiguous inclusion criteria of the study population, in which supraventricular arrhythmia (SVA) or atrial tachycardia was often grouped with AF and atrial flutter [1,16,19,24]. AF and atrial flutter are different from SVA or atrial tachycardia in pathophysiology that lies behind them and treatment goals [38-40]. The goal of treatment for AF and atrial flutter might be rhythm conversion to improve cardiac output and preventing thromboembolic events [32-34,41-47]. However, a lack of information concentrating on AF and atrial flutter makes it difficult to decide whether clinicians should do any interventions for the arrhythmia.

The aim of this review is to summarize relevant information concentrating on new-onset AF in non-cardiac critically ill patients with respect to epidemiology, prevention, and treatment.

## Methods

### Data sources and searches

We conducted a PubMed search in June 2014 using the following MeSH and keyword terms: “atrial fibrillation” or “atrial flutter” or “(atrial or supraventricular) and (tachycardia or tachyarrhythmia or arrhythmia)” and “intensive care” or “critical care” or “critically ill” or “critical illness.” Only articles published in English were considered for this systematic review.

### Study selection

The primary author (TY) and the secondary author (TF) independently screened titles and abstracts of articles identified by the search and checked in full-text level for concise selection. We included studies describing the epidemiology, prevention, and treatment of new-onset AF and atrial flutter during ICU stay in non-cardiac adult patients. Exclusion criteria were studies without description of settings as ICU, studies that focused exclusively on a perioperative period of a specific procedure, studies mainly on patients with heart diseases and cardiac surgery, and studies that did not exclude patients with past history of AF. We also excluded reviews and commentaries that did not contain original information and reports that were published only in abstract form and that provided no clear definition of patients or arrhythmias studied. Reports on patients with severe sepsis and septic shock but that were not specifically mentioned to be conducted in the ICU were included because those patients were highly expected to have been in the ICU. While atrial flutter was included because of its risk of thromboembolic events similar to AF, SVA, which was not specified further, was excluded in this review as it was out of our scope as mentioned above. Disagreement was adjudicated through consensus of the two reviewers.

As this study of a systematic review did not contain human research, approval of an institutional review board was waived.

### Data extraction and quality assessment

The primary author (TY) extracted information for the systematic review and undertook quality assessment. Selected studies were divided into three categories according to the extracted information: etiology/outcome, prevention, and treatment. The methodological quality of selected studies was described according to the Grading of Recommendations Assessment, Development and Evaluation (GRADE) system [48]. Extracted data to evaluate the risk of bias, indirectness, and imprecision were as follows: details of diagnosis of AF, allocation concealment, blinding, detailed description of conversion from AF to sinus rhythm, applicability of each study population, reported outcome, and sample sizes. We scored all items and classified into four categories from very low to high as overall quality.

## Results

**Figure 1:**
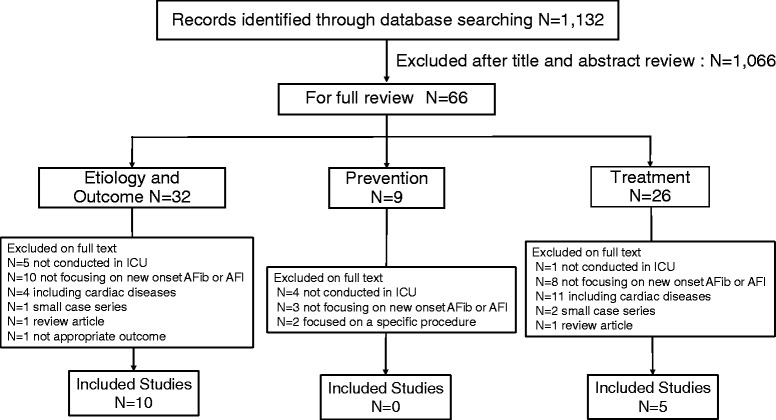
Flow chart of studies selected in the systematic review. AFib: atrial fibrillation, AFl: atrial flutter.

Figure 1 shows the flow chart of studies selected in the systematic review. We identified 1,132 citations and 66 studies were evaluated in full-text review. Thirty-two of the 66 studies related to etiology/outcome, nine studies related to prevention, and 26 studies related to treatment. After full-text-level selection, we included in this review ten studies on etiology/outcome [26,49-57] and five studies on treatment [51,53,55,58,59]. On the other hand, there was no study related to prevention that could be included in this review. Although there were two studies on prevention performed in the ICU, they focused on patients after lung resection surgery and thus were excluded [60,61]. Three studies were related to both etiology/outcome and treatment [51,53,55]. In the study by Balser et al., they screened SVA and included only those who did not convert to sinus rhythm after an adenosine challenge to rule out reentrant atrioventricular nodal rhythm and ventricular tachyarrhythmia, so that this study was included in this review [58].

Table 1 shows the overall characteristics of included studies. Of the ten studies for etiology/outcome, three studies were retrospective studies and the others were prospective. Of the five studies for treatment, there was only one randomized controlled trial (RCT). The number of patients included in each study varied from 29 to 49,082. Although one study was not performed in the ICU, it met our criteria as it was of septic shock patients [54].

**Table 1:** **Overall characteristics of included studies**

**Author, year [ref]**	**Study design**	**No. of patients**	**Setting (patients)**	**Outcome**
Bender JS, 1996 [49]	Prospective observational	206	Surgical ICU	Etiology
Seguin P, 2004 [50]	Prospective observational	453	Surgical ICU	Etiology/outcome
Seguin P, 2006 [51]	Prospective observational	293	Surgical ICU (trauma)	Etiology/outcome treatment
Arora S, 2007 [52]	Prospective observational	61	Mixed ICU	Etiology/outcome
Christian SA, 2008 [26]	Retrospective observational	272	Mixed ICU (sepsis)	Etiology/outcome
Meierhenrich R, 2010 [53]	Prospective observational	629 (50 septic shock)	Surgical ICU	Etiology/outcome treatment
Walkey AJ, 2011 [54]	Retrospective population-based cohort	49,082	Acute care hospitals (severe sepsis)	Etiology/outcome
Kanji S, 2012 [55]	Retrospective observational	3,081	Three mixed ICUs	Etiology treatment
Tongyaoo S, 2013 [56]	Prospective observational	247	Medical ICU	Etiology/outcome
Makrygiannis SS, 2014 [57]	Prospective observational	133	Mixed ICU	Etiology
Balser JR, 1998 [60]	Randomized controlled	64^a^	Surgical ICU	Treatment
Sleeswijk ME, 2008 [61]	Prospective observational	29	Mixed ICU	Treatment

^a^Number of patients with supraventricular tachyarrhythmia (not focusing on AFib and AFl).

Table 2 shows the methodological quality of included studies. Overall quality of evidence was low or very low due to their observational study designs, small sample sizes, flawed diagnosis of new-onset AF, and the absence of mortality evaluation.

**Table 2:** **Methodological quality of included studies**

**Author, year [ref]**	**Main outcome**	**Study design**	**Risk of bias**	**Indirectness**	**Imprecision** ^**a**^	**Overall quality**
Bender JS, 1996 [49]	Etiology/outcome	Prospective (0.5), observational (2)	AF diagnosis unclear (−1)	Mortality not evaluated (−0.5)		Very low (1)
Seguin P, 2004 [50]	Etiology/outcome	Prospective (0.5), observational (2)				Low (2.5)
Seguin P, 2006 [51]	Etiology/outcome	Prospective (0.5), observational (2)		Only trauma patients (−0.5)		Low (2)
Seguin P, 2006 [51]	Treatment	Prospective (0.5), observational (2)		Absence of definition for conversion time (−1)	*N* = 16 (−1)	Very low (0.5)
Arora S, 2007 [52]	Etiology/outcome	Prospective (0.5), observational (2)			*N* = 61 (−1)	Very low (1.5)
Christian SA, 2008 [26]	Etiology/outcome	Observational (2)	Retrospective diagnosis of AF (−1)	Only septic patients (−0.5)		Very low (0.5)
Meierhenrich R, 2010 [53]	Etiology/outcome	Prospective (0.5), observational (2)				Low (2.5)
Meierhenrich R, 2010 [53]	Treatment	Prospective (0.5), observational (2)		Unknown efficacy of each treatment (−1)	*N* = 49 (−1)	Very low (0.5)
Walkey AJ, 2011 [54]	Etiology/outcome	Observational (2)	Retrospective diagnosis of AF (−1)	Only septic patients (−0.5)	*N* = 49,082 (+2)	Low (2.5)
Kanji S, 2012 [55]	Etiology/outcome	Observational (2)	Retrospective diagnosis of AF (−1)		*N* = 2,895 (+1)	Low (3)
Kanji S, 2012 [55]	Treatment	Observational (2)	Retrospective diagnosis of AF (−1)	Unknown efficacy of each treatment (−1)		Very low (0)
Tongyaoo S, 2013 [56]	Etiology/outcome	Prospective (0.5), observational (2)				Low (2.5)
Makrygiannis SS, 2014 [57]	Etiology/outcome	Prospective (0.5), observational (2)		Mortality not evaluated (−0.5)		Low (2)
Balser JR, 1998 [60]	Treatment	Randomized (4)	Open label (−0.5), Allocation concealment unclear (−0.5)	Mortality not evaluated (−0.5)	*N* = 64 (−1)	Very low (1.5)
Sleeswijk ME, 2008 [61]	Treatment	Prospective (0.5), observational (2)		Mortality not evaluated (−0.5)	*N* = 29 (−1)	Very low (1)

^a^Sample size: *N* > 10,000 (+2), *N* ≥ 1,000 (+1), *N* < 100 (−1).

Table 3 shows incidences of AF and severity scores of the study population. In four studies performed in the surgical ICU, the incidence of new-onset AF was similar, ranging from 5.3% to 7.8% [49-51,54]. The incidence varied from 4.5% to 29.5% in four studies performed in the mixed ICU [26,52,55,57]. In a single study performed in the medical ICU, the incidence of AF was 13.8% [56]. When focused on sepsis and severe sepsis, new-onset AF occurred in 5.9% of septic patients [26,54], which was similar to that in surgical ICU populations. However, the incidence jumped up to 46.0% in patients with septic shock [53]. Most studies reported a severity of illness with Simplified Acute Physiology Score (SAPS) II [62] or Acute Physiology and Chronic Health Evaluation (APACHE) II score [63]. Severity scores of patients with new-onset AF were reported to be higher than those without new-onset AF in eight studies, in four of which the difference was statistically significant [50-52,56].

**Table 3:** **Incidences of new-onset atrial fibrillation and severity scores of the study population**

**Author, year [ref]**	**Setting patients**	**Incidence of AF (no.)**	**Severity score**	**Mean score** ^**a**^
Bender JS, 1996 [49]	Surgical ICU	6.8% (14/206)	NA	NA
Seguin P, 2004 [50]	Surgical ICU	5.3% (24/453)	SAPS II	45 vs. 31 (*p* = 0.0001)
Seguin P, 2006 [51]	Surgical ICU (trauma)	5.5% (16/293)	SAPS II	47 vs. 31 (*p* < 0.001)
Arora S, 2007 [52]	Mixed ICU	29.5% (18/61)	APACHE II	25.4 vs. 20.0 (*p* = 0.005)
			SAPS II	47.8 vs. 37.1 (*p* = 0.001)
Christian SA, 2008 [26]	Mixed ICU (sepsis)	5.9% (16/272)	APACHE II predicted survival	49% vs. 55% (*p* = 0.36)
Meierhenrich R, 2010 [53]	Surgical ICU	All patients 7.8% (49/629)	SAPS II	NA
		Septic shock 46.0% (23/50)		31 vs. 30^b^ (*p* = 0.12)
Walkey AJ, 2011 [54]	Acute care hospitals (severe sepsis)	5.9% (2,896/49,082)	Number of organ failures	3.11 vs. 3.08^b^ (*p* = 0.42)
Kanji S, 2012 [55]	Three mixed ICUs	4.5% (139/3,081)	APACHE II	22.6^c^
Tongyaoo S, 2013 [56]	Medical ICU	13.8% (34/247)	APACHE II	24.4 vs. 17.0^d^ (*p* < 0.001)
Makrygiannis SS, 2014 [57]	Mixed ICU	15.0% (20/133)	APACHE II	17.9 vs. 15.7 (*p* = 0.16)

*AF* atrial fibrillation, *APACHE II* Acute Physiology and Chronic Health Evaluation II, *SAPS II* Simplified Acute Physiology Score II, *NA* not available.

^a^Atrial fibrillation vs. no atrial fibrillation.

^b^Median.

^c^No information for APACHE II in no new-onset AF patients provided.

^d^All types of supraventricular tachyarrhythmia included.

Table 4 shows a list of risk factors for new-onset AF. Five studies reported risk factors for new-onset AF, all of which used multivariate analyses to extract risk factors [50-52,55,58]. All studies reported advanced age in common, two studies reported right heart catheter [50,55], and two studies reported sepsis [52,58] as independent risk factors. Medication with β-blocking agents was shown not to have any relationship with new-onset AF in three studies [50-52]. In turn, one study reported that medication with calcium channel blockers had a positive relationship with the incidence of new-onset AF [50]. Severity of illness was evaluated in four studies, three of which found severity of illness (SAPS II ≥ 30, shock, three or more regions traumatized, SIRS, APACHE II score ≥20) to have high odds ratios for new-onset AF with statistical significance.

**Table 4:** **Risk factors for new-onset atrial fibrillation**

**Category**	**Risk factor**	**OR (95%CI)**	**Reference**
Demographics	Age > 65 years	7.0 (2.0–24.6)	[58]
	Age ≥ 40 years	6.3 (1.4–28.7)	[51]
	Age ≥ 75 years	4.79 (1.16–19.8)	[52]
	Age, per 10 years	1.52 (1.47–1.56)	[54]
	Advanced age	1.04 (1.01–1.07)	[50]
	Female sex	0.83 (0.76–0.90)	[54]
	Hispanic (white as reference)	0.58 (0.50–0.63)	[54]
	Black (white as reference)	0.67 (0.58–0.78)	[54]
Past history	Calcium channel blockers	3.87 (1.18–12.7)	[50]
	Prior stroke	1.64 (1.35–2.01)	[54]
	Metastatic/hematologic malignancy	1.23 (1.09–1.39)	[54]
	Obesity	1.20 (1.03–1.40)	[54]
	Hypertension	0.88 (0.81–0.95)	[54]
	Diabetes mellitus	0.82 (0.75–0.90)	[54]
Severity	SAPS II ≥ 30	11.6 (1.3–103.0)	[51]
	Shock	6.77 (2.17–21.1)	[50]
	SIRS	4.4 (1.2–16.1)	[51]
	APACHE II score ≥ 20	3.90 (1.00–16.7)	[52]
Organ failure	Respiratory failure	2.81 (2.48–3.19)	[54]
	Congestive heart failure	1.61 (1.41–1.83)	[54]
	Hematologic failure	1.50 (1.34–1.68)	[54]
	Renal failure	1.40 (1.26–1.56)	[54]
	Per organ failure	1.12 (1.05–1.19)	[54]
	Acidosis	0.87 (0.77–0.97)	[54]
Trauma	Blunt thoracic trauma	16.8 (4.00–71.2)	[50]
	Three or more regions traumatized	6.2 (1.8–21.4)	[51]
Infection	Sepsis	6.5 (2.0–21.1)	[57]
	Sepsis at admission	4.87 (1.24–18.8)	[52]
	Abdominal infection	1.77 (1.59–1.97)	[54]
	Fungal infection	1.59 (1.27–2.00)	[54]
	Respiratory tract infection	1.27 (1.14–1.40)	[54]
	Skin or soft tissue infection	1.33 (1.14–1.55)	[54]
	Gram-positive bacteria	1.29 (1.18–1.55)	[54]
	Primary bacteremia	1.17 (1.02–1.36)	[54]
	Urinary tract infection	0.89 (0.81–0.99)	[54]
Intervention	Catecholamine use	5.7 (1.7–19.1)	[51]
	Pulmonary artery catheter	5.46 (1.84–16.2)	[50]
	Right heart catheterization	2.25 (1.87–2.70)	[54]

*OR* odds ratio, *APACHE II* Acute Physiology and Chronic Health Evaluation II, *CI* confidence interval, *SAPS II* Simplified Acute Physiology Score II, *SIRS* systemic inflammatory response syndrome.

**Figure 2:**
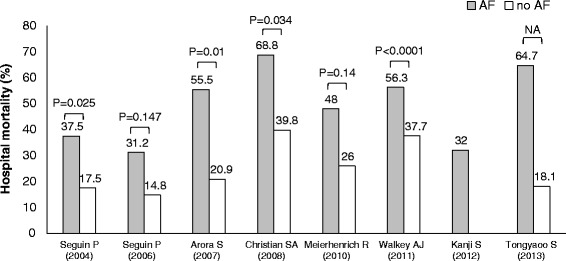
Reported hospital mortality rates in patients with and without atrial fibrillation. AF: atrial fibrillation, gray bar: AF patients, white bar: non-AF patients.

Table 5 and Figure 2 show outcomes of new-onset AF. Among eight studies reporting outcomes of AF patients, new-onset AF was associated with increased ICU length of stay in four studies [26,50,51,53] and increased hospital length of stay in one study [50]. Three studies reported the incidence of stroke that ranged from 0% to 5.9% [54-56]. A large cohort study of severe sepsis showed a significantly higher incidence of stroke in the new-onset AF group compared with the non-AF group (2.6% vs. 0.7%, *p* < 0.0001) [54]. Only one study referred to systemic therapeutic anticoagulation, in which 16% of the patients with new-onset AF were treated [55]. None of the study patients with new-onset AF had a stroke during the course of AF. Hospital mortality rates in new-onset AF groups were higher than those of no-AF groups in seven studies, in which four studies found statistical significance (Figure 2) [26,50,52,54]. However, none of the studies evaluated the impact of new-onset AF on mortality with multivariate analysis including severity scores.

**Table 5:** **Outcomes of patients with new-onset atrial fibrillation**

**Author, year [ref]**	**ICU LOS (days, mean)**	**Hospital LOS (days, mean)**	**Stroke (%)**
Seguin P, 2004 [50]	16 vs. 7 (*p* = 0.0001)	34 vs. 22 (*p* = 0.009)	NA
Seguin P, 2006 [51]	22 vs. 10 (*p* < 0.001)	32 vs. 25 (*p* = 0.259)	NA
Arora S, 2007 [52]	10 vs. 4^a^ (NS)	47 vs. 22^a^ (NS)	NA
Christian SA, 2008 [26]	17.7 vs. 8.3 (*p* = 0.003)	32.1 vs. 28.5 (*p* = 0.68)	NA
Meierhenrich R, 2010 [53]	30 vs. 17^ab^ (*p* = 0.017)	NA	NA
Walkey AJ, 2011 [54]	NA	NA	2.6 vs. 0.7 (*p* < 0.0001)
Kanji S, 2012 [55]	10^ac^	24^ac^	0
Tongyaoo S, 2013 [56]	NA	NA	5.9 vs. 2.0^d^

Atrial fibrillation vs. no atrial fibrillation.

*AF* atrial fibrillation, *LOS* length of stay, *NA* not available, *NS* not significant.

^a^Median.

^b^Data focusing on septic shock patients.

^c^Data with new-onset AF alone.

^d^ *p* value not provided.

Table 6 shows the efficacy of treatment for new-onset AF. There were five studies on treatment for new-onset AF [51,53,55,58,59]. Balser et al., the only RCT among the five studies, evaluated a conversion rate from AF to sinus rhythm within 2 h after starting administration of either esmolol or diltiazem [58]. There was no statistical difference in the conversion rate between the two study drugs (59.1% vs. 27.3%, *p* = 0.067). Kanji et al. reported a conversion rate within 24 h after various interventions. They did not describe details of combination of rhythm control therapies (direct current cardioversion, amiodarone, sotalol) and rate control therapies (β-blockers, calcium channel blockers, or digoxin, alone or in combination), so that the conversion rate could not be directly comparable to each intervention [55]. Sleeswijk et al. evaluated an efficacy of a protocol of magnesium sulfate (MgSO_4_)-amiodarone step-up scheme, which was MgSO_4_ administration followed by additional administration of amiodarone if no conversion to sinus rhythm or no reduction of the ventricular rate under 110 beats/min was attained. Although this study was not a comparative study, the reported conversion rate was high (93.1%) [59]. Meierhenrich et al. reported 85.7% of a conversion rate with various interventions, including direct current cardioversion, amiodarone, digitalis glycosides, and β-blockers, alone or in combination [53]. ICU mortality was significantly higher in patients who failed to be cardioverted than those who were converted (71.4% vs. 21.4%, *p* = 0.015).

**Table 6:** **Efficacy of treatment for new-onset atrial fibrillation**

**Author, year [ref]**	**Study design**	**Severity score**		**Observation period**	**Intervention**	**Conversion rate**		**Hospital mortality (%)**
Balser JR, 1998 [58]	Randomized controlled	APACHE III	59	Within 2 h	Esmolol	59.1% [20/34]	*p* = 0.067	31
			65		Diltiazem	27.3% [6/22]		38
Seguin P, 2006 [51]	Prospective observational	SAPS II	47	10 ± 10 h	DC	100% [3/3]	NA	31.2
					Amiodarone	100% [4/4]		
					Digoxin	100% [1/1]		
					No intervention	100% [4/4]		
Sleeswijk ME, 2008 [59]	Prospective observational	APACHE II	19	Within 24 h	MgSO_4_-amiodarone step-up scheme	93.1% [27/29]	NA	37.9
Meierhenrich R, 2010 [53]	Prospective observational	SAPS II	31^a^	NA	DC (17/49)	85.7% [42/49]^c^	NA	48^ad^
			34^b^		Amiodarone (36/49)			23^bd^
					Digitalis (31/49)			
					β-Blockers (25/49)			
Kanji S, 2012 [55]	Retrospective observational	APACHE II	22.6	Within 24 h	DC^e^	26.9% [7/26]	NA	32
					Amiodarone^f^	87.4% [90/103]		
					Sotalol	100% [2/2]		
					Rate control	75% [21/28]		

*AF* atrial fibrillation, *DC* direct current cardioversion, *LOS* length of stay, *MgSO*_*4*_ magnesium sulfate, *NA* not available.

^a^New-onset AF, no septic shock.

^b^New-onset AF and septic shock.

^c^The efficacy of each intervention was unknown because of a combination of these interventions.

^d^Sixty-day mortality.

^e^Eighteen of 26 had received amiodarone.

^f^Amiodarone alone.

## Discussion

In this systematic review, we found that the incidence of new-onset AF in non-cardiac critically ill patients widely ranged. Meierhenrich et al. reported a high rate of AF among septic shock patients (46.0%) [53]. Arora et al. reported an incidence of 29.5% in their mixed ICU, where more than 95% of patients were mechanically ventilated and nearly 30% received renal replacement therapy [52]. Excluding these two studies with exceptionally severe cases, the incidence of new-onset AF for non-cardiac critically ill patients in the general ICU was from 4.5% to 15.0%. Postoperative AF had been reported to occur in 15% to 50% of patients with cardiac surgery [32,33] and 10% to 42% with thoracic surgery [18-24], suggesting that these two specific cohorts might have a higher incidence of AF compared with the general ICU population. Since the risk factor with the highest odds ratio for new-onset AF was blunt thoracic trauma (Table 4, odds ratio = 16.8), a directly invasive procedure or trauma on the thorax might significantly contribute to the occurrence of new-onset AF in critically ill patients. Nonetheless, the incidence range in our review (5% to 15.0%) is higher than previously thought (4% to 9%) [3], possibly due to the aging population and development of more complex surgical procedures.

Severity of illness (APACHE II, SAPS II, SIRS, shock), organ failures, and sepsis were all reported as risk factors of AF in multiple studies. A subgroup of septic shock patients also had a markedly high incidence of new-onset AF (46%) [53]. These findings suggest that systematic inflammation might have a role in triggering AF in critically ill patients. Studies in cardiac surgery and the general population also reported that systemic inflammation could trigger AF, and there have been clinical trials using anti-inflammatory agents, such as colchicine and corticosteroid, to prevent or treat AF [10,12-15,64]. The effect of anti-inflammatory agents for AF in critically ill patients is unknown and further studies are needed. In the well-known CORTICUS study (hydrocortisone therapy for patients with septic shock), the incidence of AF or any types of arrhythmias was unfortunately not reported [65].

Although studies in cardiac surgery patients showed that β-blockers had a prophylactic effect for postoperative AF [29-35], none of the studies in this review showed a significant relationship between β-blockers and occurrence of new-onset AF. On the other hand, previous treatment with calcium channel blockers was reported as a risk factor of new-onset AF in one study, in which 70% of the patients received calcium channel blockers for hypertension [50]. Although hypertension was a major risk factor for AF in general populations, the authors of the study did not take a past history of hypertension into consideration in their analysis. As the authors discussed in their article, the association between previous medication with calcium channel blockers and new-onset AF was not conclusive. Walkey et al. reported a negative association between a previous history of hypertension and new-onset AF among septic patients (odds ratio = 0.88) [54]. As this study was a retrospective observational study using disease classification codes in in-patient databases, there might have been a reporting bias (e.g., less frequent coding of chronic comorbidities). To solve this uncertainty, future studies for risk factors of AF in the critically ill should include both a past history of hypertension and previous antihypertensive treatment, especially β-blockers and calcium channel blockers.

Walkey et al. presented increased risks of stroke associated with new-onset AF in septic shock patients [54]. In a recent prospective observational study on SVA, CHADS2 and CHA2DS2-VASc predicted SVA-related arterial thromboembolic events in critically ill patients, and the most accurate threshold was a CHADS2 score of 4 or higher [66]. On the other hand, Kanji S et al. reported that only 16% of new-onset AF patients in their study were given systemic anticoagulation and the incidence of stroke was 0% during the course of AF [55]. They also reported that 9% of patients with systemic anticoagulation had a bleeding event that required blood transfusion. Although ACC/AHA/ESC practice guidelines recommended anticoagulation for new-onset AF of more than 48 h of duration in the general population [33], it is currently unclear whether non-cardiac critically ill patients with new-onset AF should be anticoagulated to prevent arterial thromboembolic events. Intensive care physicians should carefully weigh the risks and benefits of anticoagulation when a critically ill patient develops new-onset AF.

As for AF prevention, we excluded two RCTs because of focusing on patients after lung resection, although they were conducted in the ICU [60,61]. The first study compared preemptive amiodarone and MgSO_4_ administration with a control, and the second study compared acebutolol and diltiazem with placebo. In the first study, both amiodarone and MgSO_4_ administration decreased new-onset AF and shortened ICU length of stay and hospital length of stay [60]. In the second study, although patients on acebutolol had a numerically lower incidence of new-onset AF than those on diltiazem and placebo, the difference did not reach statistical significance (*p* = 0.067) [61]. In addition, among the seven prevention studies we excluded from our systematic review, there were two RCTs for prevention of new-onset AF, one after transthoracic esophagectomy [67] and one after pulmonary resection [64]. In both studies, patients who received amiodarone had significantly less postoperative AF. In another study for patients undergoing pneumonectomy, prophylactic MgSO_4_ administration was also reported to reduce the incidence of SVA [68]. As for AF treatment, amiodarone and MgSO_4_-amiodarone step-up therapy achieved a conversion rate of more than 90% in mixed ICU patients who developed new-onset AF [59]. Although these findings suggest that amiodarone, MgSO_4_, or their combination might be effective to prevent or treat new-onset AF in specific groups in the ICU, more studies are needed to evaluate the effect of these drugs in the general ICU population.

This review has several limitations. First, we included only 12 studies and their quality of evidence was generally low. Since AF has a worse prognosis compared with other SVAs and AF in the general population and needs specific attention on anticoagulation, we strictly limited to studies that had focused on new-onset AF and excluded studies on SVA [43,44,69]. For example, three of four studies included in a previous systematic review on treatment for new-onset AF in non-cardiac critically ill patients were excluded in our review because these three studies also included patients with SVA [36]. We could not find any clinical trials on treatment for new-onset AF that were published after this systematic review. Second, there was a strong heterogeneity between included studies with respect to selected population and definitions of variables that had been measured, so that we could not derive conclusive statements especially on the effect of prevention and treatment of new-onset AF from this review.

## Conclusions

In this systematic review, we found that new-onset AF occurred in 5%–15% of general non-cardiac critically ill patients. Patients with new-onset AF had poor outcomes compared with those without AF. Despite the high incidence of new-onset AF in the general ICU population, currently available information for AF, especially for management (prevention, treatment, and anticoagulation), is quite limited. Further research is urgently needed to improve our understanding of new-onset AF in critically ill patients.

## Abbreviations

AF: Atrial fibrillation
SVA: Supraventricular arrhythmia
RCT: Randomized controlled trial
SAPS: Simplified Acute Physiology Score
APACHE: Acute Physiology and Chronic Health Evaluation
MgSO_4_: Magnesium sulfate
